# High Diversity of Bradyrhizobial Species Fix Nitrogen with Woody Legume *Spartocytisus supranubius* in a High Mountain Ecosystem

**DOI:** 10.3390/microorganisms11051244

**Published:** 2023-05-09

**Authors:** Laura Pulido-Suárez, Jesús Notario del Pino, Francisco J. Díaz-Peña, Adolfo Perdomo-González, Águeda M. González-Rodríguez, Milagros León-Barrios

**Affiliations:** 1 Department of Biochemistry, Microbiology, Genetics and Cellular Biology, University of La Laguna, 38200 San Cristóbal de La Laguna, Spain; 2 Department of Animal Biology, Soil Science and Geology, University of La Laguna, 38200 San Cristóbal de La Laguna, Spain; jnotario@ull.edu.es (J.N.d.P.); fjdiazpe@ull.edu.es (F.J.D.-P.); apgonzalez@ull.edu.es (A.P.-G.); 3 Department of Botany, Ecology and Plant Physiology, University of La Laguna, 38200 San Cristóbal de La Laguna, Spain; aglerod@ull.edu.es

**Keywords:** bradyrhizobia, legume (Genisteae), rhizobia, symbiosis symbiovars, high mountain soils

## Abstract

The symbiosis between rhizobia and legumes is of pivotal importance in nitrogen-poor ecosystems. Furthermore, as it is a specific process (most legumes only establish a symbiosis with certain rhizobia), it is of great interest to know which rhizobia are able to nodulate key legumes in a specific habitat. This study describes the diversity of the rhizobia that are able to nodulate the shrub legume *Spartocytisus supranubius* in the harsh environmental conditions of the high mountain ecosystem of Teide National Park (Tenerife). The diversity of microsymbionts nodulating *S. supranubius* was estimated from a phylogenetic analysis of root nodule bacteria isolated from soils at three selected locations in the park. The results showed that a high diversity of species of *Bradyrhizobium* and two symbiovars can nodulate this legume. Phylogenies of ribosomal and housekeeping genes showed these strains distributed into three main clusters and a few isolates on separate branches. These clusters consist of strains representing three new phylogenetic lineages of the genus *Bradyrhizobium*. Two of these lineages belong to the *B. japonicum* superclade, which we refer to as *B. canariense*-like and *B. hipponense*-like, as the type strains of these species are the closest species to our isolates. The third main group was clustered within the *B. elkanii* superclade and is referred to as *B. algeriense*-like as *B. algeriense* is its closest species. This is the first time that bradyrhizobia of the *B. elkanii* superclade have been reported for the canarian genista. Furthermore, our results suggest that these three main groups might belong to potential new species of the genus *Bradyrhizobium*. Analysis of the soil physicochemical properties of the three study sites showed some significant differences in several parameters, which, however, did not have a major influence on the distribution of bradyrhizobial genotypes at the different locations. The *B. algeriense*-like group had a more restrictive distribution pattern, while the other two lineages were detected in all of the soils. This suggests that the microsymbionts are well adapted to the harsh environmental conditions of Teide National Park.

## 1. Introduction

Legumes (family Fabaceae) are the third largest plant family, comprising almost 20,000 species [1]. One of their most important characteristics is their ability to establish a symbiosis with nitrogen-fixing rhizobia, an advantage that allows them to thrive in nitrogen-poor soils and be pioneer colonizers. It is also one of the main reasons for their widespread distribution in diverse habitats. Given the large size and diversity of this family, its almost 740 genera are arranged in over 40 different tribes. One of the largest is tribe Genisteae, comprised of mostly woody legumes distributed preferentially in temperate climates [2].

Rhizobia belonging to the genus *Bradyrhizobium* are the predominant microsymbionts of most species from the Genisteae tribe [2]. Even so, other rhizobia able to effectively nodulate these plants have also been reported, such as *Mesorhizobium* [3], *Ensifer* (syn. *Sinorhizobium*) [4,5,6], *Neorhizobium* and *Rhizobium* [7,8]. Moreover, some other species from non-classical rhizobial genera have also been sporadically isolated from root nodules of several genista, such as *Phyllobacterium* in *Cytisus*, *Adenocarpus*, *Retama* and *Genista* [8,9], *Ochrobactrum* in *Cytisus* and *Lupinus* and *Microvirga* in *Lupinus* [10,11].

The Canary Islands are a volcanic archipelago located in the eastern Atlantic Ocean, next to the Western Sahara coast. Despite the small surface of this territory (7500 km^2^), it is home to an enormous richness of endemic plant species, 45 of which belong to the Fabaceae family [12]. These endemic legumes are distributed in a great diversity of environments ranging between the coast and the mountainous peaks. Studies on the symbiosis of rhizobia with canarian Genisteae are so far limited to a few species of endemic shrubby legumes of foraging interest [13,14]. In these previous works, it was found that these legumes are mainly nodulated by *Bradyrhizobium canariense* and, to a lesser extent, by other genospecies of this genus [14,15,16]. The present study addresses the symbiosis of rhizobia with a shrubby genista of high ecological value, *Spartocytisus supranubius* (L.f Christ ex G. Kunkel) (syn. *Cytisus supranubius*) [17], an endemic species to the high mountain ecosystem of Tenerife. On this island, most of this alpine ecosystem is located within Teide National Park (N.P.), and *Spartocytisus supranubius*, locally known as “Retama del Teide” (Teide broom), is its most representative species. 

The survival of this endemism is currently at stake due to a combination of abiotic and biotic factors [18,19,20,21]. In fact, its population is decreasing to such an extent that it has been recently proposed as a vulnerable species in accordance with the IUCN criteria of threatened species [22]. The nature and type of soils in this area constitute additional cause for concern, as they are mostly shallow and scarcely developed (thus lacking B-type horizons), coarse-textured and poor in organic carbon and nitrogen [23,24]. As such, the available surface for the establishment of vegetation is, to a certain extent, limited. Biological Nitrogen Fixation (BNF), the result of the symbiosis between rhizobia and legumes, plays a key role in plant productivity and soil fertility, and is pivotal for the maintenance of the whole ecosystem [25,26]. The nitrogen-fixing symbiosis of *S. supranubius* with rhizobia maintains a higher soil nitrogen (N) content in the rhizospheric soil and eventually acts as the main N input in this ecosystem [16,20,27]. Given the key role of BNF in this ecosystem, it is of great interest to study the presence, abundance, diversity and symbiotic characteristics of the rhizobia capable of effectively nodulating *S. supranubius* in its natural habitat.

The distribution of microorganisms, including rhizobia, is influenced more by the soil properties [28,29] than by other factors usually related to the distribution of plants and animals, such as climate [30,31]. Although, for rhizobia, a large variety of abiotic and biotic factors are known to affect their distribution over large areas, the host plant, soil texture and soil pH have generally been recognized as determinant properties at a local level [28,29,32,33,34,35], regardless of whether other parameters, such as salinity and nutrient concentration, can also be relevant to certain bacterial groups [29,36,37]. Bacterial communities can be shaped by the soil conditions, even at short distances (<10 km) between sampling points [28]. In the case of rhizobia, their symbiotic effectiveness is often adapted to local environmental factors [29], highlighting the importance of knowing which strains are present and dominate a particular location.

In a previous study, the characterization of the rhizobia-nodulating *Spartocytisus supranubius* in a single location of Teide N.P showed that, in agreement with previous results for other canarian genista, these rhizobia belong to the genus *Bradyrhizobium* and are closely related to the *B. canariense* species [16]. However, the fact that two strains are clustered in a distant second lineage raised the question of whether a greater diversity of rhizobia could nodulate broom plants. In this work, three locations of the park were explored with the main objective of unraveling the diversity of the rhizobial species able to nodulate *Spartocytisus supranubius*. In addition, we aimed to analyze the distribution and abundance of these rhizobia in these soils, as well as the influence of the soil properties on the distribution of genotypes.

## 2. Materials and Methods

### 2.1. Site Description and Sampling

This study was carried out in Teide N.P. in Tenerife (Canary Islands), which extends from 2000 to 3718 m a.s.l. Together with the highest peaks of La Palma, it constitutes the only case of an alpine ecosystem in the Canary Islands. The park lies within the dry pluviseasonal supramediterranean bioclimatic belt and its climate is characterized by high daily and annual temperature fluctuations and irregular precipitation, mostly occurring in winter, and with a long drought period in summer [38]. Three sites in the north-eastern sector of Teide N.P. were selected for this study: Fasnia, Chiqueros and Llano de Maja (Figure S1). These locations were selected due to their well-established natural population of *Spartocytisus supranubius* broom shrubland. Fasnia (UTR 28R351796E/3130005N, 2180 m a.s.l) is located at the NE of Fasnia volcanoes. Llano de Maja (UTM 28R348721E/3130005N) is a flat area, surrounded by gentle slopes, at 2283 m a.s.l. Chiqueros is the southernmost site sampled, at 28R347555E/3129667N and 2094 m a.s.l. 

Soil was sampled from the three locations in June and October 2019. The soil samples were collected using aseptic spades and kept in sterile plastic bags. Prior to their use for microbiological purposes, the soil samples were sieved through a 3 mm mesh to remove larger pebbles and kept at 4 °C until their use in the next two–three days. For the physicochemical analysis, the samples were air-dried, passed through a 2 mm mesh and stored at room temperature until analyzed within the next two-to-six weeks.

The experiment followed a simple random sampling design, with six replicates per location.

### 2.2. Rhizobia Isolation

Germinated seedlings inoculated with the sampled soils were used to estimate the number of *S. supranubius*-rhizobia, as well as their diversity. Given the hardness of the seed coatings, seeds of *S. supranubius* had to be scarificated prior to germination. The seeds’ coats were softened in boiling water for 3 min, followed by a chemical treatment (35 min in sulphuric acid 96%). The seeds were then surface sterilized (ethanol 96%, 20 s; 50%-diluted commercial bleach NaClO 35 g/L, 10 min) and washed 6 times with distilled, sterile water to remove any remaining chemicals, to be finally germinated in agar (1%)–water plates for 5 days in the dark [16]. Seedlings with roots of similar lengths were planted in conical plastic pots (5 cm × 5 cm × 15 cm) filled with sterile vermiculite. The Most Probable Number (MPN) method was used to determine the number of viable rhizobia in the soils [39]. In addition, root nodules produced by the trap plants in the MPN experiment were also used for rhizobial isolation. For the MPN experiment, seedlings were inoculated with 1 mL of tenfold dilution of the different types of soil (combining 3 soil replicates) and grown in a growth chamber (16 h light/8 h darkness, room temperature) watered with N-free solution for 8 weeks [40]. The number of rhizobia was estimated from the nodulation data, as described in Somasegaran and Hoben [39]. Functional reddish nodules were collected and used for the isolation of root nodule bacteria, following standard procedures [41]. The nodules were surface sterilized, as previously described for seeds, and then crushed in 50µL distilled water, inoculated in yeast-mannitol agar (YMA) [41], and incubated at 28 °C until growth was observed. In case more than one type of colony was observed, the culture was purified until obtaining pure cultures.

### 2.3. Phylogenetic Analysis

A selection of isolates representing the three locations was used for taxonomic identification. Their 16S rRNA and two housekeeping genes, *glnII* and *recA*, were sequenced. Strains were selected based on their isolation site and the colony aspect, so that all locations and types of bacteria were equally represented.

The 16S rRNA gene was sequenced using the universal primers fD1 and rD1 [42]. For the housekeeping genes, the pair of primers used were glnII12F-glnII689R and recA63F-recA555R, respectively, as described in [43]. In addition, the nodulation gene *nodC* was amplified using primers nodCI and nodCF in a selection of strains representing the main phylogenetic lineages according to the concatenated and 16S rRNA phylogeny [44]. The primers and PCR conditions used can be seen in Table S1. Bacterial genomic DNA was extracted from 5–7-day YMA cultures using a commercial kit (AquaPure Genomic DNA isolation kit, BioRad). The extracted DNA was quantified in a NanoDrop 1000 Spectrophotometer and normalized to 10ng DNA/µL [16]. The phylogenetic analyses were performed using MEGA X [45]. Phylogenetic trees were assembled with MEGA X using the Neighbour-Joining (NJ) and Maximum-Likelihood (ML) methods [46,47]. NJ trees were built using the Kimura 2-parameter method. For ML trees, the best fitting-evolutionary model of nucleotide substitutions for each type of sequence was determined using MEGA X. The robustness of the tree topology was calculated using bootstrap (BS) analysis with 1000 (NJ) and 500 (ML) replications. Complete sequences of 16S rRNA, *glnII*, *recA* and *nodC* were also retrieved from the type strains of the species used as reference (NCBI: www.ncbi.nlm.nih.gov, accessed on 1 November 2022 and the EzBiocloud database https://help.ezbiocloud.net/ezbiocloud-16s-database/, accessed on 1 November 2022). The closest type strains were used to assemble the phylogenetic trees. The accession numbers of our strains are listed in Table S2. 

### 2.4. Reinfection Tests

A subset of 15 representative strains (Table S3) from the three main 16S rRNA phylogenetic branches, including representatives of the two symbiovars, were further tested to check their ability to reinfect the original host and nodule appearance. *Bradyrhizobium canariense* BTA-1^T^ type strain was also included in the analysis as it is the main species found in nodules of canarian woody genista. Seedlings (6–8 replicates for each strain) were inoculated with 1 mL of the correspondent strain at 2 MacFarland standard turbidity. The seed availability of this protected species limited a larger plant test with more strains and replicates. Both the germination and plant growth were as described in Section 2.2. Plants were harvested to check nodulation six weeks after inoculation. 

### 2.5. Soil Physicochemical Analysis

Some relevant soil properties were determined in the gathered samples. For the particle size distribution, density measurements were made in soil suspensions previously dispersed through overnight rotation shaking with a 0.36% (w:v) Na_6_[PO_3_]_6_ solution to quantify the silt and clay contents, whereas the sand contents were measured after passing the suspensions using suitable metallic sieves (0.2 mm and 0.05 mm mesh), according to Boyoucos et al. [48]. The soil pH and electrical conductivity (EC) were determined in the soil:water saturated paste extracts [49]. The soil oxidizable carbon (SOC) was determined after wet oxidation with 1N K_2_Cr_2_O_7_ and 98% sulphuric acid and further quantification of Cr^+3^ ion through UV-VIS spectrophotometry at 630 nm using a calibration curve with sucrose as C standard [50]. The total nitrogen (TN) content was determined using the Kjeldahl method [51]. Soil-available phosphorous (P) was extracted with a 0.5M NaHCO_3_ solution (1:20 w:v ratio) and measured using UV-VIS spectrophotometry, using the blue molybdenum complex and L-ascorbic acid as the reducing agent [52,53]. Soil available ammonium (NH_4_^+^) was extracted with 2 M KCl, as described in [54], and determined colorimetrically at 405 nm with the Nessler reagent [55]. All spectrophotometric measurements were made in 96-well microplates and determined in a microplate reader Labtech LT-4000 (Labtech, Italy).

### 2.6. Statistical Analysis

Statistics calculations were performed using the IBM SPSS Statistics V21.0 software package. The significance level for all tests was set to *p* = 0.05. To check for differences in the soil characteristics among the study sites, a one-way ANOVA and LSD or T2 Tamhane post-hoc test were performed for normally distributed (Kolgomorov-Smirnov test) and homocedastic (Levene test) variables. Otherwise, non-parametric (i.e., Kruskal- and Tukey-type) tests were used instead. Principal component analysis (PCA) was performed using the ‘stats’ package, R version 4.1.2, using the soil parameters’ standard scores [56], and plotted using the ‘autoplot’ function from the R ggfortify 0.4.14 package [57]. PCA of the soil physicochemical properties was conducted on 72 samples and was based on the calculated component loading values for EC, pH, SOC, N, P, NH_4_^+^, clay, silt and sand content.

## 3. Results

### 3.1. Soil Characteristics

The results of the soil analysis are shown in Table 1. The three locations under study follow a texture gradient from the Chiqueros (higher sand content) to the Fasnia samples (vice versa). When the average particle size distributions are plotted in a texture diagram (Figure S2), the Chiqueros and Maja samples appear within the sandy loam domain, whereas the Fasnia samples, on average, possess a loamy texture. In addition, remarkable differences can be observed in the soil oxidizable C (SOC) contents, as the respective average values for the Fasnia and Maja sampling locations (11.2 and 11.4 g C·kg^−1^) are twice as high as that measured at Chiqueros (5.5 g C·kg^−1^ soil). As expected, such differences are entirely related to the total N contents, for which the same ratios can be observed when comparing the Maja and Fasnia samples, on one hand, and the Chiqueros samples, on the other. To a lesser degree, such differences are also applicable to the mineral N (as NH_4_^+^) and available P contents, whereas the differences in the soil pH and EC values are less evident; however, the statistical analysis allowed us to distinguish the Chiqueros samples (on average closer to 7) from the rest as they were slightly more acidic.

Nevertheless, the results for the soil samples studied here agree with those reported in recent works conducted along the Teide N.P. that have shown low levels of soil nutrients in this particular ecosystem, especially C, N and P, whose respective biogeochemical cycles are closely linked [24,58,59].

The Chiqueros samples differed from the rest to a greater extent in other characteristics, being the most oligotrophic and least acidic soils. This differentiation might be related to the nature of the soil parental material. A query to the available spatial data on lithological types in Tenerife Island (available from the Canarian Spatial Data Infrastructure website via WMS at https://www.idecanarias.es/, accessed on 1 November 2022) reveals that Chiqueros soils have developed over basaltic and trachybasaltic lava beds, whereas both Fasnia and Maja soils result from basaltic pyroclastic materials, where mineral weathering rates are expected to be faster than in massive volcanic bedrocks [59,60,61]. In addition, some of the aforementioned relief features at the studied areas could also explain these differences in the soil properties, particularly in the case of Maja, as they would eventually favor the accumulation of fine material carried by overland water flow towards topographically depressed areas.

**Figure 1:**
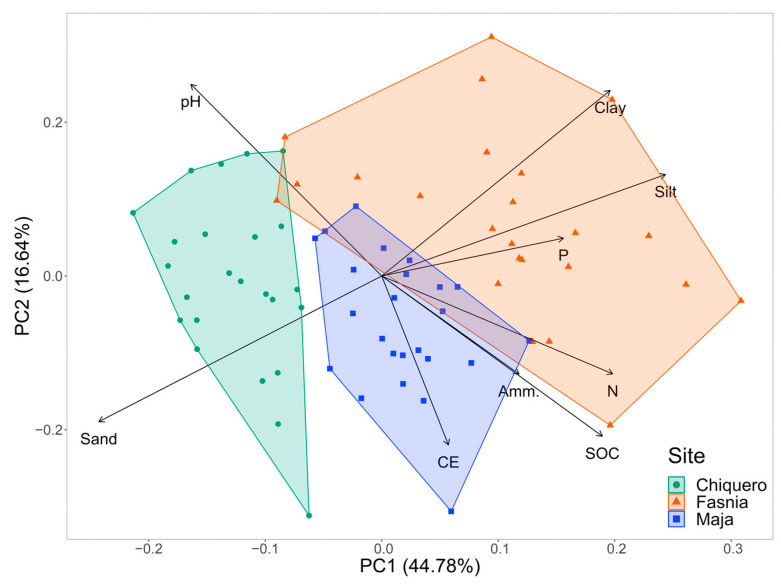
Principal Component Analysis (PCA) of the soil physicochemical properties according to the sampling location.

The multivariate analysis (PCA) confirmed these observations (Figure 1). In this analysis, the percentage of variance explained with two components was 61.42%, a rather high value, indicating that the conclusion derived from the two-dimensional figure is representative of the true sample ordination. The first component (PC1) is clearly related to the soil texture (sand vs. [clay + silt] contents). The polygons corresponding to the three locations studied align on this axis in a rather similar way to the textural diagram plot (Figure S2). In PC1, the silt content showed the greatest positive weight (score of 0.44), whereas the sand content had the greatest negative weight (score of −0.44). The second component (PC2) seems to depend on the oxidizable C content and related soil properties. The soil organic matter inversely correlates with the soil pH, given its acidic chemical reaction, and is a nutrient source via mineralization processes, thus explaining the correlation with the soil electrical conductivity. Soil N, organic or mineral, very often relates to soil organic C. Finally, the vector for the available P contents shows a tighter relationship with the silt + clay values, rather than with the soil organic C, probably because of the typical reactivity of volcanic soils towards orthophosphate ion, which lead to its partial immobilization via sorption processes, even in vitric (i.e., C-poor, non-allophanic) soils [61]. For PC2, the pH had the highest positive weight (score of 0.46), while the EC had the greatest negative weight (score of −0.44).

### 3.2. Estimation of Rhizobia Number

The rhizobial populations were estimated at the three study sites. The highest number of rhizobia was found in the soils from Fasnia (8.6 × 10^3^ rhizobia/g soil), followed by Maja (3.8 × 10^3^ rhizobia/g soil) and Chiqueros (3.2 × 10^3^ rhizobia/g soil). These results could be correlated with the nutrient content, as it was in the poorest soils of Chiqueros that the lowest number of rhizobia was recovered. However, it cannot be ruled out that the influence of other soil characteristics, such as the pH and texture, could also influence the population size of these rhizobia.

### 3.3. Phylogenetic Diversity of the Rhizobia Nodulating Spartocytisus supranubius

A total of 38 strains were selected for 16S rRNA sequencing to elucidate the phylogenetic relationships of the *S. supranubius* microsymbionts. The majority were slow-growing bacteria with an appearance compatible with *Bradyrhizobium*, the usual microsymbiont of Genisteae. A few fast- or moderate-growing bacteria were sporadically isolated from some nodules, but were not capable of reinfecting the original legume and were discarded for further analysis.

#### 3.3.1. Ribosomal Phylogeny

**Figure 2:**
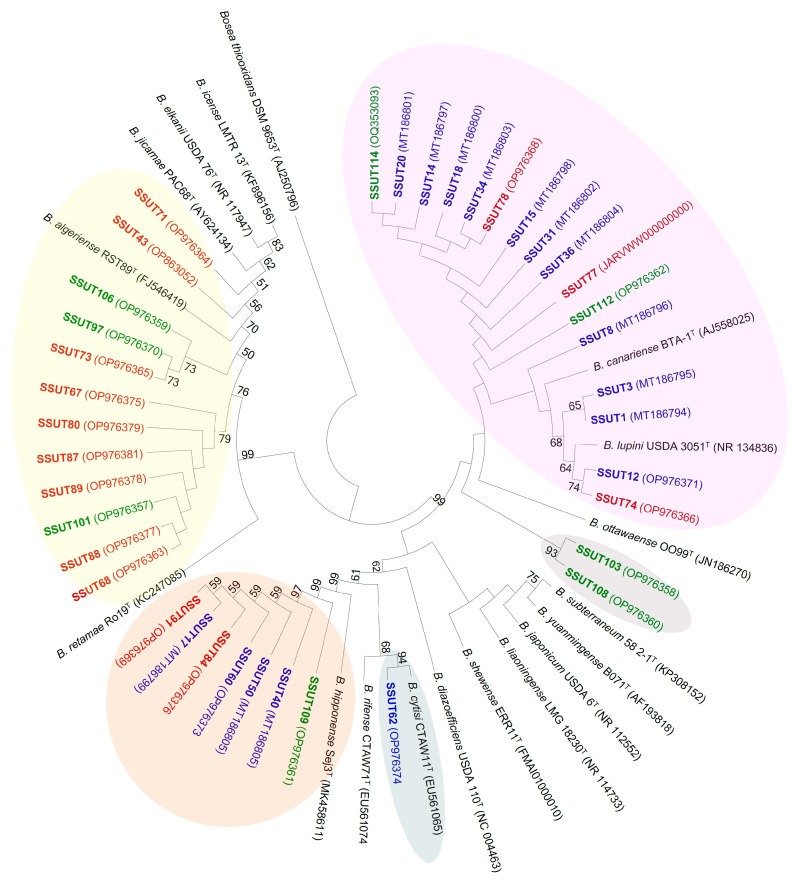
Neighbour-Joining (kimura 2-p) phylogenetic tree based on the 16S rRNA gene (1255 bp) of *Spartocytisus supranubius* bradyrhizobia and the closest reference type strains. The origin of the isolates is specified by the color. Red: isolated from Maja; green, Chiqueros; blue, Fasnia. Circles indicate the clustering of the strains. Numbers at the nodes are NJ supports from 1000. Only BS > 50% are indicated. Accession numbers are shown in parenthesis.

The results from the 16S rRNA gene phylogeny showed that *S. supranubius* is nodulated by the rhizobia of the genus *Bradyrhizobium*. Both NJ and ML gave similar clusterings. A simplified NJ circular tree, containing only the closest species, is presented in Figure 2 (Figure S3 presents a more extended tree, showing relationships with more distant species). 

Our strains showed sequence identities above 99% with several other currently described species of *Bradyrhizobium*, which is consistent with the well-established fact that highly conserved ribosomal sequences are a common characteristic in this genus [62]. Nevertheless, three main clusters could be distinguished, along with a few other isolates distributed in another two branches. Two of these clusters belong to the superclade *Bradyrhizobium japonicum* and one to *Bradyrhizobium elkanii*.

One main group, comprising 16 strains, was grouped within the *B. canariense* BTA-1^T^/*B. lupini* USDA 3051^T^ clade (no BS support). These strains shared over 99.34–100% similarities among them and 99.2–99.93% with these two sister species (with 16S rRNA gene sequences 99.66% similar). Within this big clade, four of the strains were closer to *B. lupini* (99.78–100% similar) and clustered with this species in a moderately supported subbranch (Figure 2 and Figure S3). The other 12 occupied a subbranch closer to *B. canariense* (moderately-BS-supported), with which they shared similarities in the range of 99.34–99.93%. It includes strains from the three locations, although most were isolated from Fasnia. A second main group, comprising seven strains, shared sequence similarities of 99.78–100% with *B. hipponnense* Seq3^T^ and was clustered close to this species on a well-supported branch (Figure 2). The isolation source of the strains in this cluster included representatives from the three locations. Three strains were also included in the *B. japonicum* superclade but showed different relationships. The *B. cytisi* branch included only one strain (SSUT62, isolated from Fasnia), sharing 99.93% similarity and forming a highly supported branch. The other two strains isolated from Chiqueros (SSUT103, SSUT108) showed an uncertain phylogenetic affiliation according to the ribosomal phylogeny, sharing 99.2–99.6% similarity with the species *B. canariense*, *B. shewense*, *B. subterraneum* and *B. japonicum*, but occupied a separate subbranch. 

The third major group consisted of 12 strains clustered within the *B. elkanii* superclade (Figure 2). Among all the species belonging to this big superclade, our strains have *B. algeriense* as the closest species, with which they shared sequence similarities between 99.30% and 100%. Within this clade, our isolates are differentiated into three genotypes, distributed in three subbranches. Only two isolates are on the branch containing *B. algeriense* (SSUT43 and SSUT71). A subgroup of seven (sharing 99.54–100% similarity among them) was clustered in a separated branch, with no reference strains, and shared the highest sequence similarity with *B. algeriense* (99.30–100%), followed closely by *B. erythrophlei* (99.23–99.92% similarity) and ‘*B. oropedii*’ (a not yet validly described species, 99.28–99.92%). Another subgroup of three strains (SSUT73, SSUT97, SSUT106; 99.11–99.56% similar) also had *B*. *algeriense* (99.77–99.85%), *B*. *erythrophlei* (99.32–99.77%) and *B. oropedii* (99.28–99.77%) as the closest species. However, it is interesting that these subgroups occupied two separate branches that do not include any of the already described *Bradyrhizobium*. In addition, most of these strains were isolated from Maja, some from Chiqueros and just one from Fasnia. The small differences in the percentages of the sequence similarities between all these strains produced unsupported branches, so no definitive conclusions can be drawn from this phylogeny.

#### 3.3.2. Phylogeny of the Concatenated *recA* and *glnII* Genes

**Figure 3:**
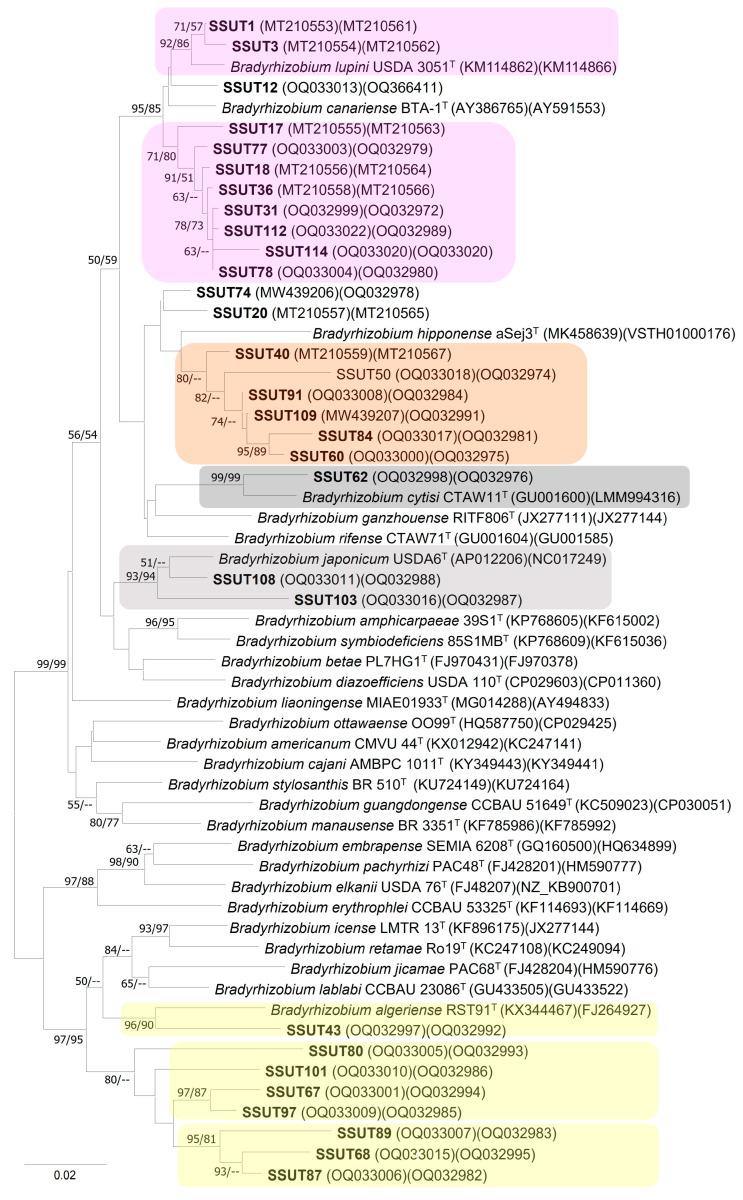
Neighbour joining (kimura 2-P) based on concatenated *glnII* (426 pb) and *recA* (320 pb) sequences showing the relationship between *S. supranubius* root nodule bradyrhizobia and the closest reference type strains of *Bradyrhizobium*. Highlighted are the lineages with BS delimited by the concatenated phylogeny. Numbers at the node are NJ and ML bootstrap values from 1000 and 500 replications, respectively. Values < 50% BS have been omitted. Accession numbers are shown in parenthesis.

The high degree of conservation of the 16S rRNA gene sequences within the genus *Bradyrhizobium* do not resolve the closely related species [15,63]. To contrast the ribosomal phylogeny, we selected *recA* and *glnII*, two of the housekeeping genes most commonly used to build phylogenies within this genus, and, as such, are sequenced in all of the described species, with a few exceptions. These two genes were sequenced for 37 of our strains, and the phylogeny of the concatenation of both genes (Figure 3) was used to better delineate our bradyrhizobial genotypes. In general terms, the results of this phylogeny agreed with the ribosomal phylogeny in showing the same main groupings. Due to the short sequence of one or both genes, seven of the originally chosen strains could not be included in the final tree. Despite their size, they could still be used to confirm their phylogenetic position, which also coincided with the 16S rRNA phylogeny. Moreover, the concatenated phylogeny better resolved the three main clades and discriminated our strains from previously described species. It also showed a few discrepancies in the relationships of some strains within the big clades.

The main clade, which contains strains close to the species *B. canariense* and *B. lupini*, showed high support (BS 95/85) (Figure 3). From the four strains that had originally grouped with *B. lupini* in the 16S rRNA tree, a close relationship with this species was only confirmed for two of the strains (SSUT1, SSUT3), which shared 98.58% similarity (in *recA*+*glnII* gene sequences) to *B. lupini*. However, this phylogeny was incongruent for the other two strains, SSUT12 and SSUT74. SSUT12 occupies a close but separate subbranch between *B. canariense* and *B. lupini*. Strain SSUT74 was placed in a distant branch from *B. canariense/B. lupini* and close to SSUT20, a strain that the ribosomal phylogeny placed close to *B. canariense* but, according to the *glnII*+ *recA* tree, is actually closer to *B. hipponense* (96.46%) than *B. canariense* (94.64%). In any case, these similarity values and the absence of BS support suggest that they do not belong to either of these two species. 

Regarding the subgroup of strains more closely related to *B. canariense* (SSUT18, SSUT 31, SSUT36, SSUT77, SSUT78, SSUT 112 and SSTU114), the concatenated phylogeny confirms its close relationship with *B. canariense* and *B. lupini.* However, they formed a well-resolved and supported subbranch within this large clade, clearly suggesting a new lineage within the clade. Close to this subgroup is now SSUT17, a strain which was closer to *B. hipponense* according to the ribosomal phylogeny; therefore, the relationships of this strain remain unresolved. 

Furthermore, the housekeeping tree resolved the strains SSUT103 and SSUT108 as close relatives of *B. japonicum* (similarity values of 94.80% and 98.16%, respectively).

Regarding the second main group of strains, which was close to *B. hipponnense* in the 16S rRNA tree, the concatenated tree resolved them in a separated branch (Figure 3). The highest similarity values for this group were with the species *B. hipponense* and *B. rifense* (92.68–95.30% and 92.39–95.72%, respectively), but the low similarity values did not allow us to discern which of the two species is the closest. However, the fact that these similarity values are less than 96% and that this well-separated branch (BS-supported) does not include any of the other previously described species is highly suggestive that these strains may represent a new lineage for the *S. supranubius* bradyrhizobia [64]. 

The third major group, in agreement with the 16S rRNA phylogeny, placed the other isolates in the *B. elkanii* superclade. Coinciding with the ribosomal tree, the concatenated phylogeny also pointed to *B. algeriense* (*recA+glnII* 94.9% similarity) as the closest species and strain. However, this phylogeny also resolved more internal divergence within this group. In particular, our strains were distributed in several subbranches with enough phylogenetic distance to even consider several species within this group. Indeed, these BS-supported subbranches do not include any recognized species, and the similarity values are lower than excepted for strains of the same species. This suggests that this group could represent new lineages for the genus *Bradyrhizobium* within the *B. elkanii* superclade. 

### 3.4. Symbiotic Phylogeny

The nodulation *nodC* gene was sequenced in a selection of 15 strains from the three main lineages previously described. The phylogeny of this symbiotic gene is presented in Figure S4. To date, 14 symbiovars (sv.) have been described within the genus *Bradyrhizobium*, but only sv. genistearum had previously been proven to be able to nodulate canarian genista. The *nodC* phylogeny resolved strains from the *B. canariense/B.lupini* lineages belonging to symbiovar genistearum, in all cases sharing > 93.6% similarity with the *nodC* sequences of the type strain, enough to consider them as being from the same symbiovar [65]. The group of strains close to *B. hipponense/B.rifense* also belonged to symbiovar genistearum (>99.07% similarity), a result consistent with those reported for the type strains of these species [66]. On the other hand, the strains in the *B. elkanii* superclade related to *B. algeriense* belong to sv. retamae, with similarity values over 99.07% with the type strains within this symbiovar [67]. The reinfection test showed that all of the strains tested from both sv. genistearum and sv. retamae were able to nodulate *S. supranubius* (Table S3). Nodulation was inferred to be effective based on the appearance of the nodules (pink nodules) and the plants (higher than the non-inoculated control plants) (Figure S5). In this reinfection experiment, no differences were observed between the plants inoculated with different rhizobial strains, neither in the number of nodules, which was low and similar (<5 nodules/plant), nor in plant height (thin and unbranched). Therefore, this assay confirmed the authentication of these rhizobia as true microsymbiont of *S. supranubius*. To conclude which of the two symbiovars has a higher nitrogen fixing efficiency will require further plant tests. 

## 4. Discussion

In this study, we showed that *Bradyrhizobium* is the rhizobial symbiont nodulating the genista *Spartocytisus supranubius*. This result is consistent with previous works that identify *Bradyrhizobium* as the main microsymbiont of the legumes of the Genisteae tribe, as summarized by Stępkowski et al. [2]. In particular, *B. canariense* has been found to be one of the main nodulating species of the Mediterranean genista legumes, including the genera *Cytisus* and *Retama*, as well as being commonly isolated from *Lupinus* in western United States [2]. Previous studies in the Canaries have also identified *B. canariense* as the main species nodulating several other genera of endemic woody genista, such as *Adenocarpus*, *Chamaecytisus*, *Teline* and *Spartocytisus* [14,15,16]. In particular, most of the isolates of *S. supranubius* characterized in those studies possessed genotypes that were compatible with bradyrhizobial strains of *B. canariense*, although two other minor bradyrhizobial genotypes, referred to as genomospecies alpha and beta, nodulating the Canary woody genista had also been detected [14,15]. According to the current phylogeny of the genus, these two genomospecies are close to the species *B. shewense* and *B. hipponense*.

In the present study, we have shown that the *S. supranubius* bradyrhizobia are in fact more diverse than previously described. Phylogenetic analysis allowed us to distinguish at least eight genotypes or lineages, none of which correspond to *B. canariense* or previously described genomospecies. Furthermore, most of our isolates, grouped in three main lineages, and the data presented here do not support their inclusion in any already recognized species, and could potentially be new species of *Bradyrhizobium*, although further taxonomic work will be needed to confirm this. 

First, the largest cluster (the *B. canariense*-like) has *B. canariense* as the closest species. However, our phylogenetic analyses suggest that these strains do not actually belong to *B. canariense* as the results are more compatible with them being a closely related new species instead. Together with *B. canariense*, the common occurrence of the species *B. lupini* among the strains nodulating the Genisteae worldwide has been previously shown [2]. It is noteworthy that two *S. supranubius*-rhizobia belonged to *B. lupini* and constitute the first record of this species for the canarian genista. However, given its scarce representation, *B. lupini* seems to be a rare microsymbiont for canarian woody genista in general, and *S. supranubius* in particular. Instead, along with *B. canariense*, we should add a potential sister species as a common microsymbiont of this endemic legume. 

The other two major lineages have also not been previously described for the canarian genista. On one hand, a group of our isolates (the *B. hipponense*-like) were clustered close to *B. hipponense* and *B. rifense. B. hipponense* was described as a symbiont of the herbaceous genista *Lupinus angustifolius* [66], while *B. rifense* was isolated from *Cytisus villosus*, a shrub legume that is also able to establish a symbiosis with *B. canariense* [68]. Our results, however, do not support the *Spartocytisus* bradyrhizobia of this group as belonging to either species, but suggest a potentially novel sister species.

On the other hand, the third main group of our *Spartocytisus*-bradyrhizobia was clustered close to *B. algeriense*, a symbiont nodulating the genus *Retama* [67], a species within the *B. elkanii* superclade. It is worth mentioning that most of the bradyrhizobia able to nodulate the genista legumes belong to the *B. japonicum* supergroup, and only a minority to the *B. elkanii* superclade [2]. This is the first time bradyrhizobia of the *B. elkanii* superclade has been isolated from the root nodules of the Canary genista. Moreover, our phylogenetic analysis suggests that these rhizobia might include new potential species. 

Regardless of these results, the confirmation of all these phylogenetic lineages as members of new species will require further work according the taxonomic criteria for novel species descriptions [69].

Finally, two *S. supranubius*-bradyrhizobia (SSUT103 and SSUT108) were closely related to *B. japonicum*. Previous studies of canarian genista had classified a few isolates from the genus *Teline* into the species *B. japonium* [13]. In our case, the housekeeping phylogeny supports the idea that SSUT108 probably belongs to *B. japonicum*, but not the SSUT103 strain.

In summary, we have shown the high diversity of *Bradyrhizobium* lineages nodulating the endemic legume *Spartocytisus supranubius* in its natural environment, which potentially belongs to at least five different species and two symbiovars. In addition, it is noteworthy that bradyrhizobia with sv. genistearum had been previously isolated from canarian woody legumes [15], but this is the first time sv. retamae has been found in these endemic species. Nevertheless, further studies are needed to evaluate the symbiotic efficacy and to establish whether there is a combination of species and symbiovar that provides more nitrogen to this legume.

The distribution of the three main clusters of *Bradyrhizobium* varied in the soils of Teide N.P., with *B. canariense*–like and *B. hipponense*-like strains detected in all three study sites (Table S2). This points to a good adaptation to different soil conditions, which results in a wide distribution in these soils. In contrast, our *B. algeriense*-like strains showed a more restrictive distribution. In particular, Fasnia soils seem less suitable for these rhizobia, as, exceptionally, only one strain was isolated in these soils. When analyzed to reveal whether the distribution of the bradyrhizobia isolates was related to the soil characteristics of each location, the results showed that texture was the most determining parameter differentiating the three studied sites. It could therefore be the most important factor influencing the rhizobia population sizes and the species’ distribution. Previous studies have highlighted the importance of soil texture [70,71], especially sand and clay content, in soil microbial communities. In fact, it has been considered to be second in importance, after the pH [72]. In our case, Fasnia had a loamy texture, different from Chiqueros and Maja, both of which had a sandy-loam texture, rich in sand (Table 1). It is noteworthy that the proportion of sand and clay, in particular, can affect the bioavailability of nutrients, such as P and water retention [32,70]. In our study, it is the site richest in silt and clay (Fasnia) that practically lacks *B. algeriense*-like strains. This suggests that texture, clay and silt, in particular, have some negative impacts on the strains from this cluster. The particular cause, however, will require further study. The particular effect of the soil texture on the bacterial diversity is not completely clear. While some authors have stated that increasing the sand content increases the bacterial richness due to a higher number of isolated hydrated microhabitats [70,72], others have observed a decrease in OTUs in coarser soils, with clay showing higher values [71]. In the family Bradyrhizobiaceae, opposite trends have also been reported. As in the present study, Hemkemeyer et al. [71] observed a depletion of *Bradyrhizobium* related to clay content, whereas Xia et al. [72] found a negative correlation with the sand content, even though alphaproteobacteria, in general, showed a positive correlation. In summary, it appears that the sensitivity to soil texture depends on each particular taxon. 

Taking the other studied soil characteristics into account, the Chiqueros samples differed to a greater extent from the rest, being the most oligotrophic and least acidic soils. The nutrient availability, particularly the pH, has been repeatedly described as one of the most determining factors in describing rhizobia distribution [29,33,34,35,37]. In our case, Chiqueros was the site with the lowest MPN, pointing to an effect of its soil characteristics in the population size. Despite this, Chiqueros hosted representatives of the three main lineages, further indicating the good adaptation of these *Bradyrhizobium* genotypes to the soil conditions at Teide N.P. 

In general, we can state that the new lineages of *Bradyrhizobium* nodulating *S. supranubius* are widely distributed in the soils of Teide N.P. These results agree with the broad geographical range of *Bradyrhizobium*, which has been related to their adaptability to different edaphic and climatic conditions, as well as to their large genomes enabling them to prosper in a wide spectrum of resources and conditions [2,35,73]. 

## 5. Conclusions

*Spartocytisus supranubius* is a shrub legume thriving in the harsh environmental conditions of the high mountain ecosystem of Teide National Park. We have shown that, in its natural habitat, this legume can be nodulated by a high diversity of species of *Bradyrhizobium* and two symbiovars. In particular, the three main groups are representative of new phylogenetic lineages of the genus *Bradyrhizobium*, and potentially three new species. Most of these genotypes were detected in the soils of the three study localities, with no clear relationship between the soil characteristics and *Bradyzobium* species distribution, suggesting that, similarly to the host legume, the microsymbionts are well adapted to the harsh environmental conditions of Teide National Park.

## Figures and Tables

**Table 1:** Average of the soil parameters measured in the three locations. Different letters indicate statistical differences according to a One-Way ANOVA (*p* < 0.05) or Kruskal-Wallis test.

Soil Parameter	Units	Fasnia	Chiqueros	Maja
EC	dS m^−1^	0.4 ± 0.1 a	0.3 ± 0.0 a	0.4 ± 0.1 a
pH	unitless	6.5 ± 0.1 b	6.8 ± 0.1 a	6.5 ± 0.1 b
SOC	g kg^−1^	11.2 ± 1.3 a	5.50 ± 0.6 b	11.40 ± 0.6 a
N	g kg^−1^	1.4 ± 0.2 a	0.6 ± 0.1 b	1.3 ± 0.1 a
P	mg kg^−1^	26.7 ± 1.7 a	15.7 ± 1.4 b	19.3 ± 1.7 b
NH_4_^+^	mg kg^−1^	44.3 ± 3.6 a	31.2 ± 2.4 b	48.8 ± 2.7 a
Sand	g kg^−1^	449.6 ± 116.5 c	759.7 ± 74.7 a	625.4 ± 55.8 b
Silt	g kg^−1^	332.9 ± 88.3 a	147.4 ± 54.1 c	263.2 ± 43.3 b
Clay	g kg^−1^	217.5 ± 49.8 a	92.9 ± 26.8 b	111.4 ± 26.9 b

## Data Availability

The datasets presented can be found in online repositories. Supplementary Table S2 includes the accession numbers of the sequences, which can be found in https://www.ncbi.nlm.nih.gov/, accessed on 1 November 2022.

## Supplementary Materials

The following supporting information can be downloaded at: https://www.mdpi.com/article/10.3390/microorganisms11051244/s1, Figure S1: Map; Table S1: Primers; Table S2: accession numbers; Figure S2: textural diagram; Table S3: reinfection test; Figure S3: 16S rRNA phylogeny; Figure S4: *nodC* phylogeny.
