# Supplementary material for: High Diversity of Bradyrhizobial Species Fix Nitrogen with Woody Legume Spartocytisus supranubius in a High Mountain Ecosystem
Source: Microorganisms. 2023 May 9;11(5):1244. doi: 10.3390/microorganisms11051244 (PMC10222482; doi:10.3390/microorganisms11051244)
Supplement: Supplementary file 1 [file microorganisms-11-01244-s001.zip › microorganisms-2338429-supplementary/Supplementary Figure S1_MAP.pdf]

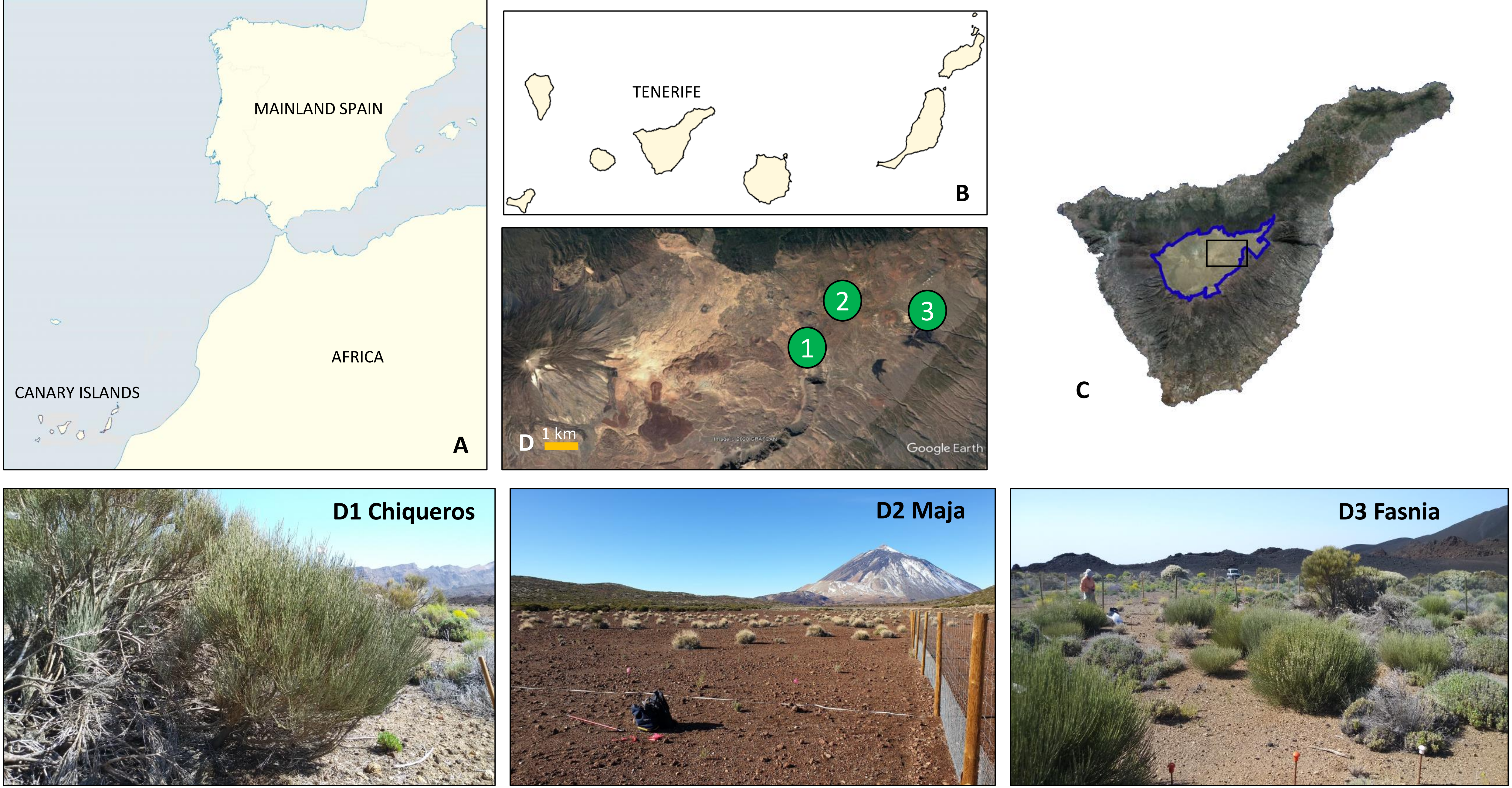

Figure S1. (A) Map of the Canary Islands with respect to mainland Spain and Africa. (B) The Canary Islands. (C) Tenerife island with the boundaries of Teide NP with the sampling area in the square. (D) The sampling points within the N.P. Panoramic pictures of the sampling sites of Chiqueros (D1), Maja (D2) and Fasnia (D3).
