# Supplementary material for: High Diversity of Bradyrhizobial Species Fix Nitrogen with Woody Legume Spartocytisus supranubius in a High Mountain Ecosystem
Source: Microorganisms. 2023 May 9;11(5):1244. doi: 10.3390/microorganisms11051244 (PMC10222482; doi:10.3390/microorganisms11051244)
Supplement: Supplementary file 1 [file microorganisms-11-01244-s001.zip › microorganisms-2338429-supplementary/Supplementary Figure S3.pdf]

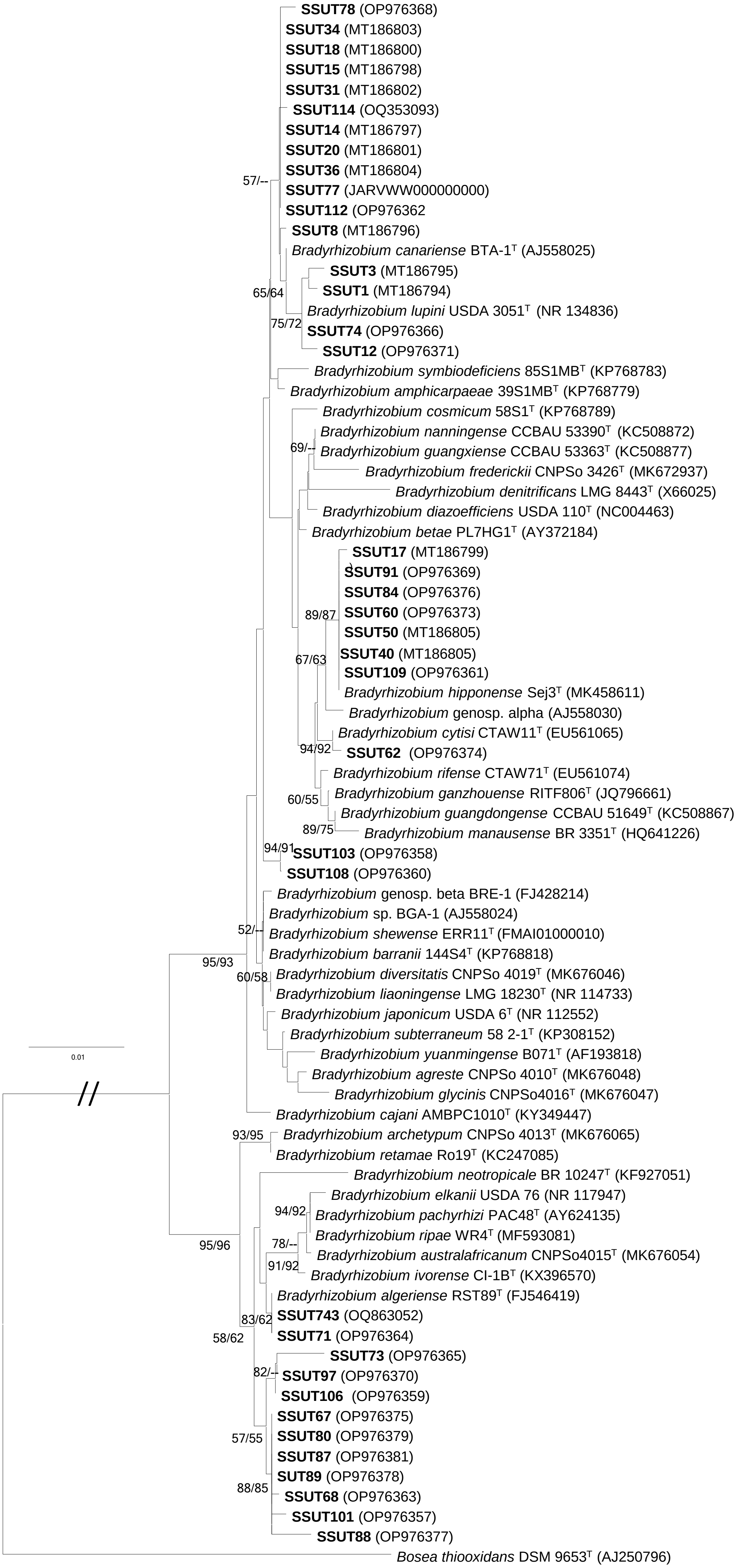

Figure S3. Neighbour-joining (kimura 2-p) phylgenetic tree based on the 16S rRNA gene (1250 pb) of *Spartocytisus supranubius* bradyrhizobia and the closest reference type strains. Numbers at the nodes are NJ and ML bootstrap supports from 1000 and 500 replications, respectively. Only BS > 50% are indicated. Accession numbers are show in parenthesis.
