# Supplementary material for: High Diversity of Bradyrhizobial Species Fix Nitrogen with Woody Legume Spartocytisus supranubius in a High Mountain Ecosystem
Source: Microorganisms. 2023 May 9;11(5):1244. doi: 10.3390/microorganisms11051244 (PMC10222482; doi:10.3390/microorganisms11051244)
Supplement: Supplementary file 1 [file microorganisms-11-01244-s001.zip › microorganisms-2338429-supplementary/Supplementary Figure S4_nodC.pdf]

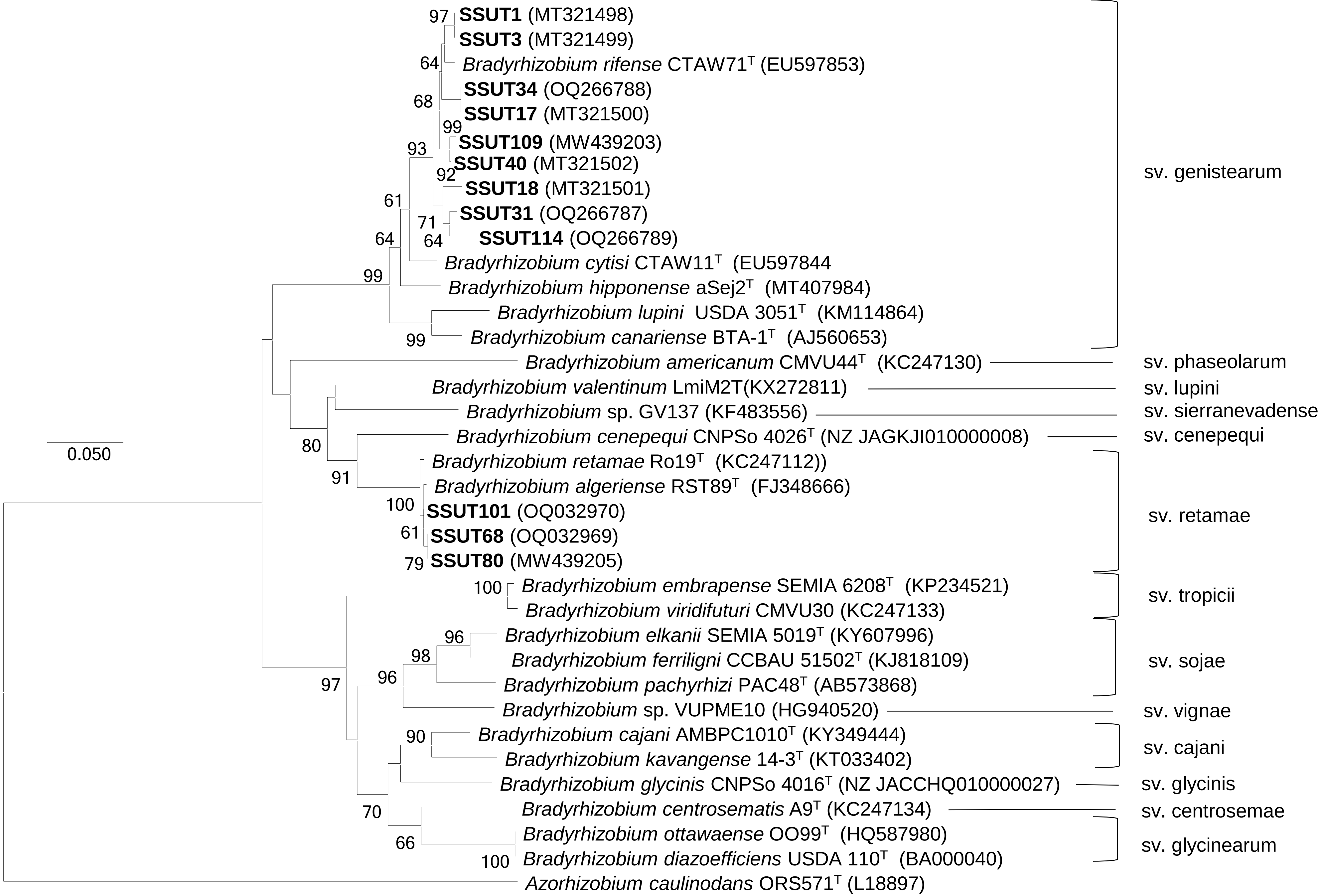

Figure S4. Neighbour-joining tree based on *nodC*sequences (486 nt) of *Spartocytisus supranubius* bradyrhizobia and reference strains. The significance of each branch is indicated by a bootstrap value (≥60%) calculated for 1000 subsets (NJ). The scale bar indicates the number of substitutions per site.
