# Supplementary material for: High Diversity of Bradyrhizobial Species Fix Nitrogen with Woody Legume Spartocytisus supranubius in a High Mountain Ecosystem
Source: Microorganisms. 2023 May 9;11(5):1244. doi: 10.3390/microorganisms11051244 (PMC10222482; doi:10.3390/microorganisms11051244)
Supplement: Supplementary file 1 [file microorganisms-11-01244-s001.zip › microorganisms-2338429-supplementary/Supplementary Table S1_primers.pdf]

Table S1. Primers and PCR conditions used in this study.

Gene	Primer	Sequence (5'-3')	PCR cycling conditions
16S rRNA	fD1<br>rD1	AGAGTTTGATCCTGGCTCTCAG<br>AAGGAGGTGATCCAGCC	3'30" 95 ºC, 30 x (1'10", 94 ºC; 40", 56 ºC; 2'10", 72 ºC), 6' 20" 72ºC
glnII	glnII12F<br>glnII689R	YAAGCTCGAGTACATYTGGCT<br>TGCATGCCSGAGCCGTTCCA	5' 95 ºC, 30x (45" 94 ºC, 1',58 ºC, 1'30", 74 ºC), 1' 74 ºC.
recA	recA63F<br>recA555R	ATCGAGCGGTCGTTCGGCAAGGG<br>CGRATCTGGTTGATGAAGATCACCAT	2' 95 ºC, 35x (45" 94 ºC, 1' 50 ºC, 1'30" 74 ºC), 1' 74 ºC.
nodC	nodCF<br>nodCI	AYGTHGTYGAYGACGGTTC<br>CGYGACAGCCANTCKCTATTG	3' 95 ºC, 35x (1' 94 ºC, 1' 55 ºC, 2' 72 ºC) 3' 72 ºC
