# Supplementary material for: High Diversity of Bradyrhizobial Species Fix Nitrogen with Woody Legume Spartocytisus supranubius in a High Mountain Ecosystem
Source: Microorganisms. 2023 May 9;11(5):1244. doi: 10.3390/microorganisms11051244 (PMC10222482; doi:10.3390/microorganisms11051244)
Supplement: Supplementary file 1 [file microorganisms-11-01244-s001.zip › microorganisms-2338429-supplementary/Supplementary Table S2_accession.pdf]

Table S2. Accession number of the genes used in this study and isolation site of the strains.

Strain	*16S*	*glnII*	*recA*	*nodC*	*Isolation site*
**SSUT1**	MT186794	MT210553	MT210561	MT321498	Fasnia
**SSUT12**	OP976371	OQ033013	OQ366411		Fasnia
**SSUT14**	MT186797				Fasnia
**SSUT15**	MT186798				Fasnia
**SSUT17**	MT186799	MT210555	MT210563	MT321500	Fasnia
**SSUT18**	MT186800	MT210556	MT210564	MT321501	Fasnia
**SSUT20**	MT186801	MT210557	MT210565		Fasnia
**SSUT3**	MT186795	MT210554	MT210562	MT321499	Fasnia
**SSUT31**	MT186802	OQ032999	OQ032972	OQ266787	Fasnia
**SSUT34**	MT186803			OQ266788	Fasnia
**SSUT36**	MT186804	MT210558	MT210566		Fasnia
**SSUT40**	MT186805	MT210559	MT210567	MT321502	Fasnia
**SSUT43**	OQ863052	OQ032997	OQ032992		Fasnia
**SSUT50**	OP976372	OQ033018	OQ032974		Fasnia
**SSUT60**	OP976373	OQ033000	OQ032975		Fasnia
**SSUT62**	OP976374	OQ032998	OQ032976		Fasnia
**SSUT67**	OP976375	OQ033001	OQ032994		Maja
**SSUT68**	OP976363	OQ033015	OQ032995	OQ032969	Maja
**SSUT71**	OP976364				Maja
**SSUT73**	OP976365				Maja
**SSUT74**	OP976366	MW439206	OQ032978		Maja
**SSUT77**	JARVWW000000000	OQ033003	OQ032979		Maja
**SSUT78**	OP976368	OQ033004	OQ032980		Maja
**SSUT8**	MT186796				Fasnia
**SSUT80**	OP976379	OQ033005	OQ032993	MW439205	Maja
**SSUT84**	OP976376	OQ033017	OQ032981		Maja
**SSUT87**	OP976381	OQ033006	OQ032982		Maja
**SSUT88**	OP976377				Maja
**SSUT89**	OP976378	OQ033007	OQ032983		Maja
**SSUT91**	OP976369	OQ033008	OQ032984		Maja
**SSUT97**	OP976370	OQ033009	OQ032985		Chiqueros
**SSUT101**	OP976357	OQ033010	OQ032986	OQ032970	Chiqueros
**SSUT103**	OP976358	OQ033016	OQ032987		Chiqueros
**SSUT106**	OP976359				Chiqueros
**SSUT108**	OP976360	OQ033011	OQ032988		Chiqueros
**SSUT109**	OP976361	MW439207	OQ032991	MW439203	Chiqueros
**SSUT112**	OP976362	OQ033022	OQ032989		Chiqueros
**SSUT114**	OQ353093	OQ033020	OQ032990	OQ266789	Chiqueros
