# Supplementary material for: High Diversity of Bradyrhizobial Species Fix Nitrogen with Woody Legume Spartocytisus supranubius in a High Mountain Ecosystem
Source: Microorganisms. 2023 May 9;11(5):1244. doi: 10.3390/microorganisms11051244 (PMC10222482; doi:10.3390/microorganisms11051244)
Supplement: Supplementary file 1 [file microorganisms-11-01244-s001.zip › microorganisms-2338429-supplementary/Supplementary Table S3_Figure S5.pdf]

Table S3. Symbiotic phenotypes of 15 representative strains from the different lineages based on the nodule appearance (pink nodules).

**Strain**[(1)]	Symbiotic phenotype
B. canariense	
SSUT18	Fix+
SSUT31	Fix+
SSUT36	Fix+
SSUT77	Fix+
SSTU78	Fix+
SSUT114	Fix+
B. lupini	
SSUT1	Fix+
SSUT3	Fix+
B. hiponense	
SSUT40	Fix+
SSUT109	Fix+
SSUT17	Fix+
B. algeriense	
SSUT67	Fix+
SSUT71	Fix+
SSUT80	Fix+
Bradyrhizobium sp.	
SSUT74	Fix+
B. canariense BTA-1$^{T(2)}$	Fix+

(1) Closest species according to the phylogenetic analysis
(2) *B. canariense* BTA-1^{T} (original host *Chamaecytisus proliferus*)

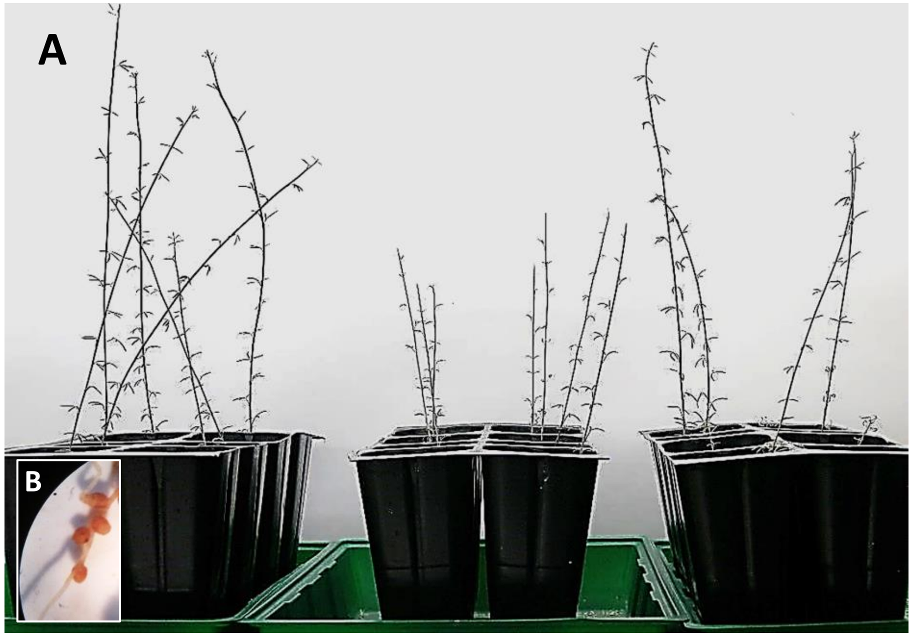

Figure S5. A) Reinfection test with SSUT1 (left), negative control (center) and SSUT18 (right). **B)** Detail of root nodules produced by SSUT1 in the reinfection test.
