# Supplementary figures and images for: High Diversity of Bradyrhizobial Species Fix Nitrogen with Woody Legume Spartocytisus supranubius in a High Mountain Ecosystem
Source: Microorganisms. 2023 May 9;11(5):1244. doi: 10.3390/microorganisms11051244 (PMC10222482; doi:10.3390/microorganisms11051244)

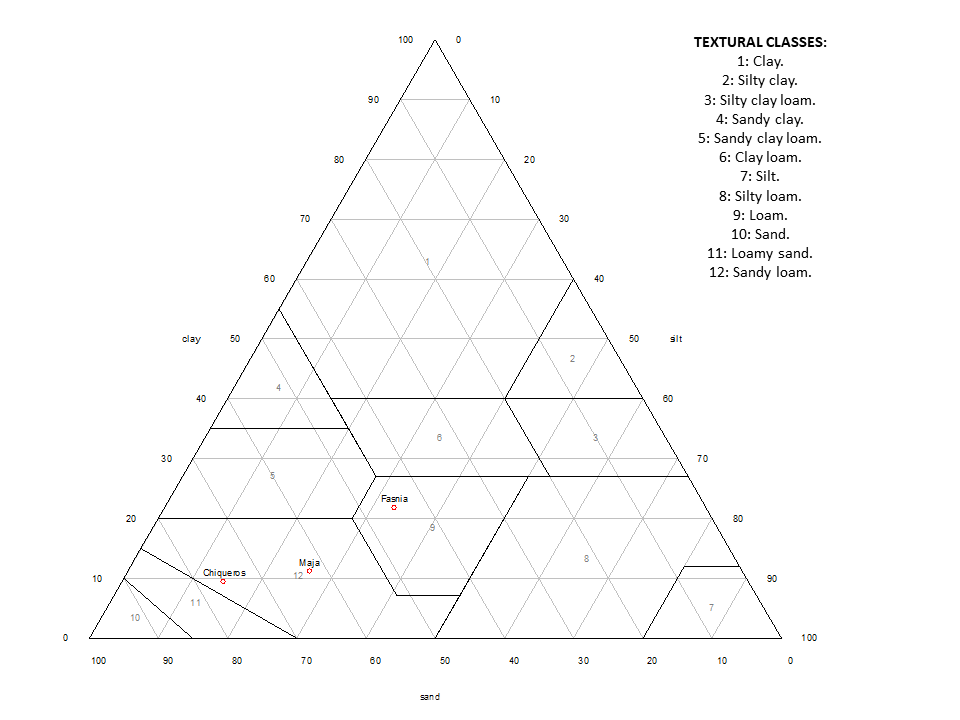

Supplement: Supplementary file 1 [file microorganisms-11-01244-s001.zip › microorganisms-2338429-supplementary/Supplementary Figure S2_textural classes.png]
